# Quebrachitol: Global Status and Basic Research

**DOI:** 10.1007/s13659-017-0120-3

**Published:** 2017-01-28

**Authors:** Dong Wang, Shuqun Zhang, Zhe Chang, De-Xin Kong, Zhili Zuo

**Affiliations:** 1 State Key Laboratory of Phytochemistry and Plant Resources in West China, Kunming Institute of Botany, Chinese Academy of Sciences, Kunming, 650201 China; 2 State Key Laboratory of Agricultural Microbiology, Huazhong Agricultural University, Wuhan, 430070 China; 3 Yunnan Key Laboratory of Natural Medicinal Chemistry, Kunming, 650201 China

**Keywords:** Quebrachitol (QCT), Structure, Biological activity, Application

## Abstract

Recently, there has been a renewed interest in the natural-products-inspired drugs. Quebrachitol (QCT) is one of naturally occurring optically active cyclitols that has now received considerable attention. Until the last decade, it came to be a starting point for the lead discovery. In this review, we had a discussion on the basic research of QCT, including its source, structure, properties, and the recent advances on its application. The biological activities and QCT-inspired leads that are potentially effective for treating human diseases were also discussed.

## Introduction

For many years, natural products have been the inspiration and an essential source for medicinal chemistry and drug discovery. An analysis of all therapeutic agents that have been approved by US FDA from 1981 to 2014 reveals that the natural products and their frameworks continuously play a profound impact on the medicinal chemistry [1]. Over 34 years, total 1562 drugs have been approved. Those classified as “Synthetic drug” category have the largest proportion, with 52%. The next is the natural product and its subdivision (“Natural product botanical drug” and “Derived from a natural product”), which account for 26%, followed by 22% being “Vaccine” or “Biological macromolecule”. Inspection of all approved drugs as shown in Table 1 demonstrates that medicinal chemists never cease exploring the utilization of natural products for drug discovery. In 2004 only, 10 of 44 approved small molecule drugs are in the categories of “Natural product”, “Natural product botanical drug” and “Derived from a natural product”. Since the 2015 Nobel Prize for Physiology or Medicine was awarded to Drs. Omura, Campbell and Prof. Tu, there has been a renascent interest in not only the natural-product-related drugs, but also in their enantioselective [2, 3]. Furthermore, the persistent use of natural products, natural product derivatives and their fragments could be anticipated, especially with the subsequent developments of computational tools, including chemo- and bioinformatics approaches [4, 5]. Isolation from natural sources is usually a primary approach to provide natural products to complete biological studies and to discover and develop new drug candidates. Quebrachitol (2-*O*-methyl-l-inositol) (QCT), a bioactive plant constituent, is one of naturally occurring optically active cyclitols that has received considerable attention as a starting point for lead discovery. Therefore, in this article we discussed the basic research of QCT, including its source, structure and properties. In addition, we gave an insight to the recent advances on its application, with a focus on its biological activities that are related to human diseases.

**Table 1:** Detailed information of all approved drugs from 1981 to 2014

	V	S/NM	S*/NM	S*	ND	NB	N	B	S	Total
1981	1	1	4	4	7	0	3	0	20	40
1982	0	0	2	0	7	0	3	5	14	31
1983	0	2	2	3	9	0	4	1	35	56
1984	0	2	2	2	14	0	1	1	22	44
1985	1	4	2	1	18	0	3	9	17	55
1986	1	3	4	0	13	0	3	6	26	56
1987	1	4	5	3	24	0	8	8	25	78
1988	0	6	6	2	16	0	6	2	24	62
1989	2	5	7	2	8	1	3	3	12	43
1990	1	3	7	0	10	0	1	6	18	46
1991	0	7	5	3	6	0	0	10	9	40
1992	1	2	2	3	13	0	2	9	14	46
1993	3	4	4	2	14	0	2	3	14	46
1994	1	5	3	2	13	0	2	8	13	47
1995	2	5	5	5	9	0	2	3	12	43
1996	2	8	8	2	7	0	1	10	12	50
1997	1	15	8	1	5	0	0	5	12	47
1998	2	10	5	1	9	0	0	9	8	44
1999	2	5	4	3	13	0	2	8	9	46
2000	7	3	7	2	12	0	1	3	12	47
2001	1	2	3	2	10	0	0	20	5	43
2002	1	8	4	2	12	0	0	3	8	38
2003	2	2	8	1	5	0	3	11	3	35
2004	2	2	3	2	7	0	0	4	4	24
2005	8	3	3	2	9	0	4	11	12	52
2006	9	10	1	3	3	1	1	10	2	40
2007	6	5	2	1	5	2	4	12	7	44
2008	6	4	4	1	8	1	0	8	6	38
2009	11	10	2	0	5	0	0	7	7	42
2010	6	6	0	0	8	0	1	7	5	33
2011	1	3	11	0	6	0	2	6	5	34
2012	9	6	9	2	6	2	2	16	8	60
2013	8	6	9	3	3	0	1	8	9	47
2014	3	11	11	1	6	2	2	18	11	65

Data from JNP [1]

*B* biological, usually a peptide or protein, *N* natural product, unmodified in structure, *NB* natural product “botanical drug”, *ND* derived from a natural product, *S* totally synthetic drug, S* made by total synthesis, but the pharmacophore is from a natural product, *V* vaccine, *NM* natural product mimic

## The Source of QCT

QCT is the byproduct of the rubber industry. The discovery history of QCT can be traced back to 1889, when it was discovered by Tanret from the quebracho bark of *Aspidosperma quebracho* for the first time. The concentration in the aqueous phase (serum) of the latex of rubber trees was reported to be 2%, and it was firstly obtained from this source by de Jong in 1906. As a chiral building block, QCT was further expanded by its availability in high concentrations from rubber tree. Then in 1932, QCT had been prepared on a larger collection by Rhodes and Wiltshire [6]. Afterwards, QCT was separated out of the red oil of Minnesota Wild Hemp [7]. The first systematic method of extracting QCT was reported by Jan van Alphen [6]. Because QCT is seldom coexisted with its isomer d-pinitol in the same plant family, isolation from natural source provides an excellent way to obtain pure compound. In addition, QCT can be found in *Allophylus edulis*, *Dipladenia martiana* and *Sapindus rarak* [8–10]. It was the active component of *Cannabis sativa* and sea buckthorn [11]. QCT could also be extracted from the South African plants enumerated hereunder: *Artemisia afra* Jacq., *Cardiospermum halicacabum* L., and *Paullinia pinata* L. Recently, QCT, identified as main constitutes in the flesh of litchi, was deemed to be the important contribution to the widely known health benefits of litchi [12].

## The Structure and Properties of QCT

QCT, a colorless crystalline compound, melts at about 192–193 °C and can be sublimed under a diminished pressure. Its boiling point in vacuum is approximately 210 °C. The spectral data and crystal structure results of QCT were discussed by Patterson et al. in 1931, Huang et al. in 1994 and Dowd et al. in 2002, respectively [13–15]. QCT crystal was placed in the monoclinic system, space group *P*2_1_, with cell parameters a:b:c = 6.60:7.15:8.658, β = 90° and Z = 2. The refractive index is n_α_ = 1.546, n_β_ = 1.552, n_ν_ = 1.572. QCT can easily dissolve in water, 100 g saturated solution containing 38 g (0 °C), 39 g (12 °C), and 70 g (l00 °C) of QCT. It is soluble in boiling alcohol, acetic acid and in pyridine, but insoluble in ether.

**Fig. 1:**
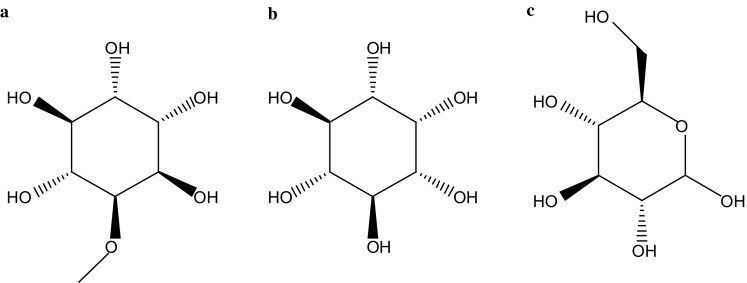
The structures of quebrachitol (**a**), inositol (**b**) and glucose (**c**)

As illustrated in the Fig. 1, QCT, which is a methyl-derivative of inositol, has similar physiological effects with inositol. In eukaryotes, inositol, usually in the form of phosphatidylinositol (PI), is a principle constituent of cell membranes and is involved in multiple pivotal transmembrane signaling pathways [16]. The structure of QCT is similar with glucose, but not in function. QCT would not enhance the blood sugar level after it was taken. It is two or three times less sweeter than cane sugar, but the edible concentration may produce colic and diarrhea [17]. Owing to this reason, Robert et al. recommended against using QCT as a sweetening agent.

## The Regulation of QCT Absorption

QCT, together with sucrose, is one of the main sugars in the latex of rubber trees. Nevertheless, the mechanisms of how QCT was absorbed remained to be elucidated in more detail. To this end, Anaïs Dusotoit-Coucaud et al. investigated and characterized the transporter of QCT from latex. A new polyol transporter, HbPLT2, was identified which acted as a plasma membrane H^+^/QCT symporter [18]. First, through screening a latex cDNA library, two full length cDNAs were isolated referred as to *Hevea brasiliensis* polyol transporter 1 and 2, respectively (HbPLT1 and HbPLT2). The two transporters exhibited 89% identity at the protein sequence levels and shared the main features of the previously cloned acyclic sugar polyol transporters. In order to examine the ability of HbPLT2 as a transporter, functional expression was carried out in yeast strains. These strains could absorb polyol but no absorption of sucrose and hexose. As a result, the phenomenon that xylitol uptake was inhibited by an excess of QCT was observed, which suggested that QCT was a substrate of HbPLT2. In conclusion, their research gave clues for the further investigations of QCT functions with regard to the physiological features of rubber trees.

## The Applications of QCT

According to above-mentioned properties, QCT could serve as the starting material of inositol or its derivatives to participate in several important pathways and may act as a potential anticancer or antidiabetic drug lead to arrest or reverse these diseases.

### Effects of QCT on Diabetes Mellitus

Diabetes mellitus (DM), currently characterized by symptoms of chronic hyperglycaemia, is a syndrome defined as a state of dysregulation of glucose homeostasis and lipid metabolism due to beta cell dysfunction or insulin resistance [19]. Although some success was achieved in lowering blood sugar, type 2 DM (T2DM) remains to be one of the widespread epidemics. Hence, there is a compelling need for discovering novel antidiabetic drugs. A growing number of studies have shown the great potential of QCT in the suppression of T2DM symptoms. Early in 1933, Robert et al. investigated the effect of QCT as a sweetening agent for diabetics [17]. Martina et al. isolated QCT from *A. edulis*, which was used to treat diabetes in the practice of local traditional medicine [8]. Yong Xue and coworkers reported their study about the effects of sea buckthorn juice and QCT on T2DM, in which db/db mice were fed with pure sea buckthorn juice or sea buckthorn juice rich in QCT for 10 weeks [20]. Their results manifested the effects of sea buckthorn juice in lowering blood sugar level, possibly concerned in the improvement of pancreas function and reversal of insulin resistance. Moreover, QCT was used to synthesize β-glucosidase inhibitors while targeting gluconeogenesis or glucose homeostasis was suggested to be a new strategy for developing more efficient DM therapeutics [21]. Thereby, QCT or its derivatives could be used as a potential antidiabetic medicine even though further research was required to improve the effectiveness.

### Effects of QCT on Inhibiting PAFR

Platelet-activating factor (PAF), a metabolite of arachidonic acid pathway, features prominently in cell proliferation, migration and angiogenesis. Results from previous studies indicated that its expression was elevated in several cancers, such as human thyroid cancer, meningiomas, breast cancer and oesophageal cancer [22–25]. Moreover, recent study showed that PAF might play pivotal roles in the progression of prostate cancer and the PAF antagonist may be potentially effective for treating castration-resistant prostate cancer (CRPC) [26].

The effects of QCT on inhibiting platelet aggregation and PAF receptor (PAFR) were reported by Moharam et al. [27]. Five compounds including QCT were first isolated from the twigs of *Mitrephora vulpina* C.E.C. Fisch and then evaluated for their inhibitory ability on PAFR binding using ^3^H-PAF. Of all the compounds tested, phylligenin and QCT stood out as the most potent inhibitors against PAFR, with IC_50_ values of 13.1 and 42.2 µM, respectively. The results of this study demonstrated the strong PAFR inhibitory effect of phylligenin and QCT. QCT could therefore be developed as a potential PAF antagonist, either alone or combined with existing drugs, for the treatment of CRPC.

### Effects of QCT on Free Radical Scavenging

The maladjustment of reactive oxygen species production and the endogenous antioxidants has been involved in the pathogenesis of several obstinate diseases, such as cancer, asthma, pulmonary hypertension, and retinopathy [28]. QCT was reported to prevent oxidative injury to diverse pathways in various pathophysiological conditions.

Peroxynitrite (ONOO^−^) scavenging and laxative effects of QCT have been reported in the previous study [29]. QCT was also proposed to have free radical scavenging ability in DPPH assay [30]. In 2006, Junior et al. pointed out that it could inhibit 6-OHDA-induced generation of nitrogen oxide in cultured mesencephalic cells [31]. The aforementioned antioxidative effect of QCT may overcome the oxidative stress and alleviate cellular dysfunction. In addition to the effect of eliminating free radicals, being a sugar-like molecule, QCT was likely to improve the mitochondrial energy metabolism so that the 6-OHDA-induced decrease of ATP could be prevented. Besides, as describing on part 3, QCT closely resembles inositol in structure, it can be used to synthesize PI, the main constituent of cell membranes. It may also lower endoplasmic reticulum stress response, which may be caused by oxidative stress. Despite the fact that its mechanisms of cytoprotection remained to be elusive, their research indicated a clear antioxidant effect of QCT.

### Effects of QCT on Gastroprotection

Studies suggested that nonsteroidal anti-inflammatory drug (NSAID) or aspirin may cause gastrointestinal complications and sometimes even death [32, 33]. In 2008, the study of Olinda et al. provided the first evidence that QCT has gastroprotective effect against acute gastric lesions [34]. According to their research, QCT dramatically prevented gastric lesions induced by ethanol and indomethacin at oral doses of 12.5 and 25 mg/kg, which was mediated at least partially by endogenous prostaglandins, nitric oxide release and $K_{ATP}^+$ channel opening. In view of mucosal erosions occurred after the administration of NSAID, QCT could save stomach cells from injuries. Their finding was in well agreement with that of Gharzouli et al., which reported that in experiments with rats, the mannitol, glucose–fructose–sucrose–maltose mixture and natural honey has the gastroprotective function against ethanol-induced gastric damage [35]. In their previous work, QCT manifested an antioxidant activity and a cytoprotective function in DPPH assay and in rat fetal mesencephalic cells, respectively. In this study, the evidence of cytoprotection against gastric mucosal damage was offered [30, 31]. These researches about gastroprotective role of QCT suggested that it could be developed as a new therapy that combat the stomach diseases arose from NSAID or aspirin use.

### Effects of QCT on Synthesis

Natural products can be used as lead compound to generate drugs with origin or novel therapeutic utility [36]. QCT furnished an alternative optically structure for the construction of compounds with specific biological activities. In 1995, Kiddle gave an excellent perspective of the utilities of QCT in asymmetric synthesis [37]. As he suggested, the particular advantage provided by QCT was that there were no synthetic limitations imposed by the presence of a hemiacetal/ketal functionality. QCT, now as a starting material, has been used to synthesize a variety of biologically active compounds, most notably the inositol, as listed in Table 2.

**Table 2:** Compounds with biological activities synthesized from QCT

Structures	Activity	Literature
	A β-glucosidase inhibitor	Akiyama et al. [44]
	An inositol 1,4,5-trisphosphate 5-phosphatase and 3-kinase inhibitor	Liu et al. [48–50]
	Bislactones, antifungal and antibacterial activities	Chida et al. [38]
	A IP_3_ 5-phosphatase inhibitor (44, 0.3 μM)	Liu et al. [49, 50]
	A phosphatidylinositol synthase inhibitor (37% inhibition)	Johnson et al. [53]
	Antifungal activity	Chida et al. [39]
	Inhibit the grow of wild type NIH 3T3 cells by 30% at 1 mM concentration	Kozikowski et al. [56]
	A partial agonist at IP_3_ receptor (EC_50_ = 1.14 ± 0.19 μM)	Liu et al. [51]
	Anti-infectious activity	Chida et al. [40]
	Threefold less potent than IP_3_ on binding and Ca^2+^ releasing activity	Kozikowski et al. [57]
	Antibiotic, antitumor and immunosuppressive activities	Barton et al. [46]
	Immunosuppressive, cytotoxic and plant grow regulator activities	Chida et al. [42]
	Antitumor, immunostimulatory, neuritogenic and growth inhibitory activities	Chida et al. [43]
	A glycosidase inhibitor	Falshaw et al. [45]
	Synthesize PI(3, 4)P_2_, PI(4, 5)P_2_ and PI(3, 4, 5)P_3_	Qiao et al. [54]
	Block the activation of the serine/threonine kinase Akt	Kozikowski et al. [55]
	Activation of IP_3_ receptor (*K*_d_ = 810 nM)	Liu et al. [52]
	Antimicrobial agents	Assis et al. [47]

#### Synthesis of Natural Products Starting from QCT

Chida and coworkers have made valuable contributions to the synthesis of biologically essential natural products, starting from QCT. Two bislactones and one natural product with antifungal activity were synthesized from QCT, described by Chida et al. [38, 39]. The stereoselective synthesis of the novel marine natural product, bengamide A, B and E starting from QCT was later reported by them [40, 41]. In 1997, Chida et al. proposed a strategy to synthesize PA-48153C, a novel 2-pyranone derivative showed potent immunosuppressive, cytotoxic and plant growth regulator activities, utilizing QCT as a chiral building block [42]. A year later, an effective synthetic pathway to synthesize novel cerebrosides was put forward, using QCT as a starting material as well [43]. Their synthetic studies emphasized the validity of QCT as a chiral starting material for the synthesis of optically active natural products.

#### Synthesis of Antibiotics and Enzyme Inhibitors Starting from QCT

Total synthesis of a variety of compounds with beneficial effects were published in succession. In 1991, Akiyama et al. reported a new strategy for the total synthesis of cyclophellitol which is a β-glucosidase inhibitor through Peterson olefination and demethylation, starting from QCT [44]. Likewise, Falshaw used QCT as a starting material to synthesize the inhibitors of β-glucosidase, which was potential effective for the treatment of various disorders and diseases such as diabetes, cancer, and AIDS [45]. Some other compounds, (−)-ovalicin and several related analogues, with antibiotic, antitumor and immunosuppressive activities were synthesized from QCT, proposed by Barton et al. [46]. The work of Assis et al. described the synthesis of inositol derivatives from QCT condensed with 2-mercaptobenzothiazole or 2-mercaptobenzimidazole, which were potential antimicrobial agents [47].

Several researches made by Liu et al. were published with regard to developing IP_3_ 5-phosphatase inhibitors. They sought to develop synthetic routines to inositol phosphates and its analogues. Total synthesis from QCT to the l-chiro-inositol 2,3,5-trisphosphate, which was a potent IP_3_ 5-phosphatase and 3-kinase inhibitor, was first reported by them [48]. They proposed the synthesis of potent IP_3_ 5-phosphatase inhibitors, l-chiro-inositol 1,4,6-trisphosphate, along with the corresponding trisphosphorothioate and evaluated their potencies [49]. In 1992, Liu et al. synthesized an inhibitor of the enzymes of 1d-myo-inositol 1,4,5-trisphosphate metabolism, using QCT as a starting material [50]. They have also synthesized an analogue of the second messenger, l-chiro-inositol 2,3,5-trisphosphorothioate, which was both a partial agonist in the release of intracellular Ca^2+^ and a lead compound for designing small molecule IP_3_ receptor antagonists [51]. Moreover, Liu et al. have designed a stable analog of IP_3_, 1d-2,3-dideoxy-myo-inositol 1,4,5-trisphosphorothioate, which could be recognized by IP_3_ receptor with a binding constant (K_*d*_) of 810 nM [52].

Besides, a number of 3-substituted 1d-myo-inositols were synthesized from QCT and evaluated as substrates for PI synthase. Johnson et al. suggested that these compounds could be used as probes for studying the PtdIns pathway [53]. Due to the importance of PIPn’s in cell signaling, Qiao et al. have designed a versatile approach to synthesize PI(3, 4)P_2_, PI(4, 5)P_2_, and PI(3, 4, 5)P_3_ from QCT [54]. In 2002, Kozikowski et al. disclosed various modified PI analogues, which could selectively inhibit serine/threonine kinase Akt without decreasing the upstream kinase PDK-1 and other downstream kinases such as MAPK [55]. Above studies suggested that these compounds, prepared by QCT, were effective lead compounds for the development of drugs designed to treat neoplastic diseases.

#### Synthesis of Inositol Derivatives Starting from QCT

Another aspect of QCT-inspired synthesis is inositols, especially on *myo*-inositol and its derivatives because of their important role in multiple cell signaling pathways, such as PtdIns(4, 5)P_2_ hydrolysis and PtdIns(3, 4, 5)P_3_ synthesis. Additionally, these compounds may have potential therapeutic value. Kozikowski et al. synthesized and studied the utilities of d-3-deoxy-3-phosphonomethyl-myo-inositol in the PtdIns-3′-kinase signaling pathway [56]. The results showed that none of these compounds significantly inhibited the growth of wild type and *v*-sis-transformed NIH 3T3 cells. Then, they synthesized the novel 3-modified IP_3_ analogues, ld-3-C-(trifluoromethyl)-myo-inositol 1,4,5-trisphosphate and 1l-chiro-inositol 1,2,3,5-tetrakisphosphate. The preliminary results suggested that the proper modification of 3-position of IP_3_ may contribute to its binding with IP_3_ receptor [57]. On the basis of studies mentioned above, Kozikowski and co-workers have found that azido inositols, synthesized from QCT could selectively inhibit the growth of *v*-sis-transformed NIH 3T3 cells. Apart from these, Almeida et al. have studied a simple route to synthesize some azido and amino inositol derivatives, starting from QCT [58]. In order to optimize the procedure and improve the yield, a modified synthesis of 1l-1,2:3,4-di-*O*-cyclohexylidene-5-*O*-methyl-chiro-inositol, which QCT was used as the original material, has been accomplished by Barrs et al. [59].

The synthetic approaches previously described have revealed that QCT has proven to be a useful optically starting point for the construction of bioactive molecules.

### Effects of QCT on Other Applications

Besides the applications mentioned above, some other effects of QCT were described. Hewitt et al. had shown that not only could QCT act as a phagostimulant for the larvae, but may also supply the *m*-inositol dietary requirements of the larvae, after conversion to *m*-inositol [60]. In 2003, Akiyama et al., developed an enantioselective Michael addition of glycine imine using QCT as a catalyst [61]. The study by Orthen et al. found that QCT has the cryoprotective effect, as it could decrease membrane damage to a similar degree as the two well-known cryoprotectants, sucrose and trehalose [62]. Another literature reported the ability of membrane stabilization of QCT, since it could improve structural and thermal stability for proteins [63].

## New Direction of QCT in Multi-target Drug Design

Drugs that blocking a single pharmacological target are often lack of effectiveness in controlling complex diseases, such as diabetes, inflammation, cancer and central nervous system disorders [64–66]. In order to treat these diseases more effectively, the concept of combination therapy was proposed. Combination drugs that using several compounds to directly interact with multiple targets have been employed [67–69]. However, a few problems would arise in the combination of different drugs, including potential drug–drug interactions and different pharmacokinetics properties, which make the drug combination very complicated. Accordingly, multi-target drugs, which could interact with multiple targets simultaneously through one compound, are prominent in the new drugs development for the treatment of complex diseases. Currently, the multi-target compound discovery techniques, namely the linker strategy, framework combination and cross virtual screening approach, have been developed. With the manifold biological activities exhibited and the aforementioned methods properly used, QCT may be potential multi-target drug lead in treating DM and cancers.

## Conclusions

In summary, we have given a brief overview of the researches and the application of QCT. Currently, the unclear macromolecular targets to act with, hampers the rational design and further optimization of QCT. According to the literature reported before, QCT exerted its biological functions, by involving IP_3_ receptor, β-glucosidase, PAFR, COX2, nitric oxide synthase, and $K_{ATP}^+$ channels. Systematic identifying the molecular targets for on-target and off-target effects of QCT, however, is still an important and currently unmet challenge. It is conceivable that through combinations of biochemical methods, genetic interactions and computational inference, the working mechanisms of QCT and its derivatives will be fully understood.
